# Nasal delivery of donepezil HCl-loaded hydrogels for the treatment of Alzheimer’s disease

**DOI:** 10.1038/s41598-019-46032-y

**Published:** 2019-07-02

**Authors:** Sitah Al Harthi, Seyed Ebrahim Alavi, Mahasen Ali Radwan, Mona Mohamed El Khatib, Ibrahim Abdullah AlSarra

**Affiliations:** 1 Department of Pharmaceutical Science, College of Pharmacy, King Saud University, Riyadh, Saudi Arabia; 2 Department of Pharmaceutical Science, College of Pharmacy, Shaqra University, Riyadh, Saudi Arabia; 3 School of Pharmacy, The University of Queensland, Woolloongabba, 4102 Australia; 4 Department of Pharmaceutics and Pharmaceutical Technology, College of Pharmacy, Egyptian Russian University, Bader City, Egypt; 5 Department of Pharmaceutics, Faculty of Pharmacy, King Saud University, Riyadh, Saudi Arabia; 6 Department of Pharmaceutics, Faculty of Pharmacy, Cairo University, Cairo, Egypt

**Keywords:** Drug delivery, Drug delivery, Drug delivery, Drug delivery, Drug delivery

## Abstract

This study aims to prepare, characterize and evaluate the pharmacokinetics of liposomal donepezil HCl (LDH) dispersed into thiolated chitosan hydrogel (TCH) in rabbits. Various hydrogels including TCH were prepared, and after characterization, TCH was selected for subsequent evaluations, due to the promising results. TCH was then incorporated with LDH prepared by reverse phase evaporation method. The hydrogel was characterized using scanning electron microscope, dialysis membrane technique, and ultra-performance liquid chromatography methods. The optimized resultant was then evaluated in terms of pharmacokinetics in an *in vivo* environment. The mean size of LDH and drug entrapment efficiency were 438.7 ± 28.3 nm and 62.5% ± 0.6, respectively. The controlled drug release pattern results showed that the half-life of the loaded drug was approximately 3.5 h. Liposomal hydrogel and free liposomes were more stable at 4 °C compared to those in 20 °C. The pharmacokinetics study in the rabbit showed that the optimized hydrogel increased the mean peak drug concentration and area under the curve by 46% and 39%, respectively, through nasal route compared to the oral tablets of DH. Moreover, intranasal delivery of DH through liposomal hydrogel increased the mean brain content of the drug by 107% compared to the oral DH tablets. The results suggested that liposomes dispersed into TCH is a promising device for the nasal delivery of DH and can be considered for the treatment of Alzheimer’s disease.

## Introduction

Alzheimer’s disease (AD) is a dysfunction of central nervous system (CNS) and causes dementia, in which patients lose their memory^1^. AD results in neuronal deterioration, given rise to memory and cognitive impairment as well as disturbance in daily activities. As a result, the patients thinking ability is disrupted, leading to psychiatric disorder^1^. In the last stages of the disease, patients require 24-h care^2^. Today, cholinergic hypothesis constitutes the basis for the development of most common therapeutics for AD treatment. According to this hypothesis, reducing the production of acetylcholine, as a neurotransmitter, leads to AD. Therefore, cholinesterase inhibitors (ChEIs) can be considered as potential targets for the treatment of AD^3^. Donepezil HCl (DH) is one of the ChEIs developed for AD treatment^4^.

DH, as a specific and reversible ChEI, increases the acetylcholine concentration in the cholinergic synapses^4^. Despite its therapeutic effects, it has various side effects, such as nausea and diarrhea, after oral administration^5^. Also, drug delivery to AD is limited, due to the blood brain barrier (BBB), restricting the brain penetration of 98 and 100% of small and large molecule drugs, respectively^6^. To overcome these issues, hydrogel-based drug delivery systems and drug delivery through nasal route seem to be appropriate.

Nasal mucosa is characterized by high vascularization, large absorptive surface area, and the rapid onset of action^4^. Moreover, this route is an alternative, non-invasive, and painless technique for circumventing the BBB and delivering therapeutics to CNS^4^.

Also, hydrogels are cross-linked 3-dimensional (3D) networks, biocompatible with biological systems, and able to absorb the high content of water^7^. Most hydrogels are bio- and muco-adhesive^8^ and improve drugs bioavailability more than conventional oral drug delivery systems through increasing the contact time between the drug and absorption site^9^. Therefore, hydrogels appear promising for brain drug delivery through nasal route^10^.

In this study, various hydrogels formulations were designed, and after characterization, thiolated chitosan hydrogel (TCH) was selected for incorporation with liposomal DH (LDH) for the intranasal delivery of DH as an anti-Alzheimer drug in rabbits. In this regard, various pharmacokinetic parameters, including the mean peak drug concentration (C_max_), time to reach C_max_ (T_max_), and area under the curve (AUC) of the drug were evaluated. LDH incorporated into TCH could considerably contribute to the development of efficacious system for DH brain delivery, due to its promising properties. This formulation is a novel approach, reported for the first time for the brain delivery of DH which might result in the development of an innovative therapy for AD treatment.

## Results and Discussion

### Characterization of hydrogels

#### Drug loading efficiency

Drug loading efficiency is critical for polymer carriers as low drug loading increases the drug cost, decreases therapeutic efficacy, and causes inappropriate release profiles, leading to the limited efficiency of drug delivery systems^11^. Therefore, improving the drug loading efficiency is important to obtain the efficient drug action. In the present study, the results showed high drug loading efficiency, confirming the potency of the method used for synthesizing DH-loaded hydrogels, in which drug entrapment efficiency for the hydrogels ranged from 79 to 95%.

#### Gel fraction

Gel fraction is the weight ratio of dry gel before and after swelling^12^. It indicates the covalent crosslinking of polymer chains^13^ and is inversely associated with the toughness of a polymeric network^14^. The poor mechanical properties of PVP limits its use for biomedical application, and its mixing with other polymers enhances its mechanical characteristics and usage as biomedical materials^15^.

**Figure 1:**
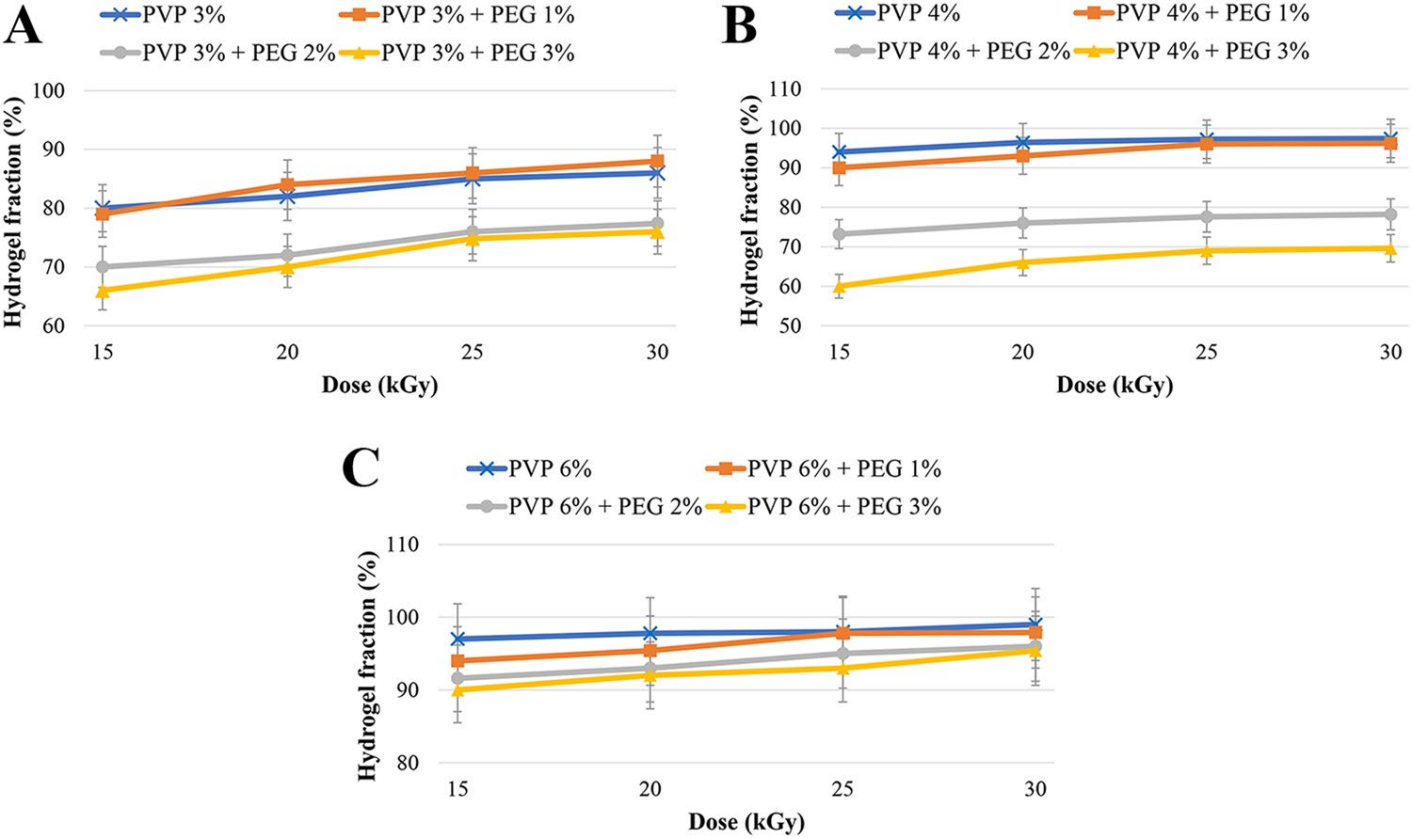
Effect of radiation dose on the hydrogel fraction in different formulations: (**A**) PVP 3% gels, (**B**) PVP 4% gels and (**C**) PVP 6% gels.

In the present study, preparing the PVP hydrogels in aqueous polymer solution and cross-linking them using gamma ray was found as a simple and effective procedure. Gel fraction data presented in Table 1 (Supplementary Information) and Fig. 1 indicated that gel fraction was generally decreased with increasing PEG concentration and decreasing radiation dose. This results from the fact that PEG functions not only as a plasticizer, but also it decreases the cross-linking degree and maintains the gelation process between PVP chains, resulting in decreasing the PVP cross-linking and further avoiding their physical interactions^16^. As PEG is an alcohol, it can function as a radical scavenger, and as a result, it can be used as an enhancer or a reducer of the hydrogel gelation based on the radiation dose. This effect can be reversed by increasing the radiation dose, which causes further cross-linking network chains and increasing the gel content^17^. Based on these facts, the difference between gel fraction values of the hydrogels prepared from only PVP (3, 4 and 6%) and hydrogels containing PEG 3% were statistically significant (P < 0.05). These results were in agreement with the results of El-Mohdy *et al*.^17^ and Kim *et al*.^18^ studies; however, in the present study, chitosan and TCH showed comparable gel fraction values (65 and 62% for chitosan and TCH, respectively). The degree of polymer cross-linking is inversely proportional with the degree of swelling^19^, and as the gel fraction increases, the lower swelling degree is achieved^20^.

#### Degree of swelling

It has been found that cross-linking density is inversely associated with the swelling degree^21^. Also, the extensibility and the elastic modulus are decreased as the swelling degree increases^22^. Moreover, a direct relationship between PEG content and the swelling degree of hydrogels has been reported as PEG blocks are hydrophilic and drive swelling^23^.

**Figure 2:**
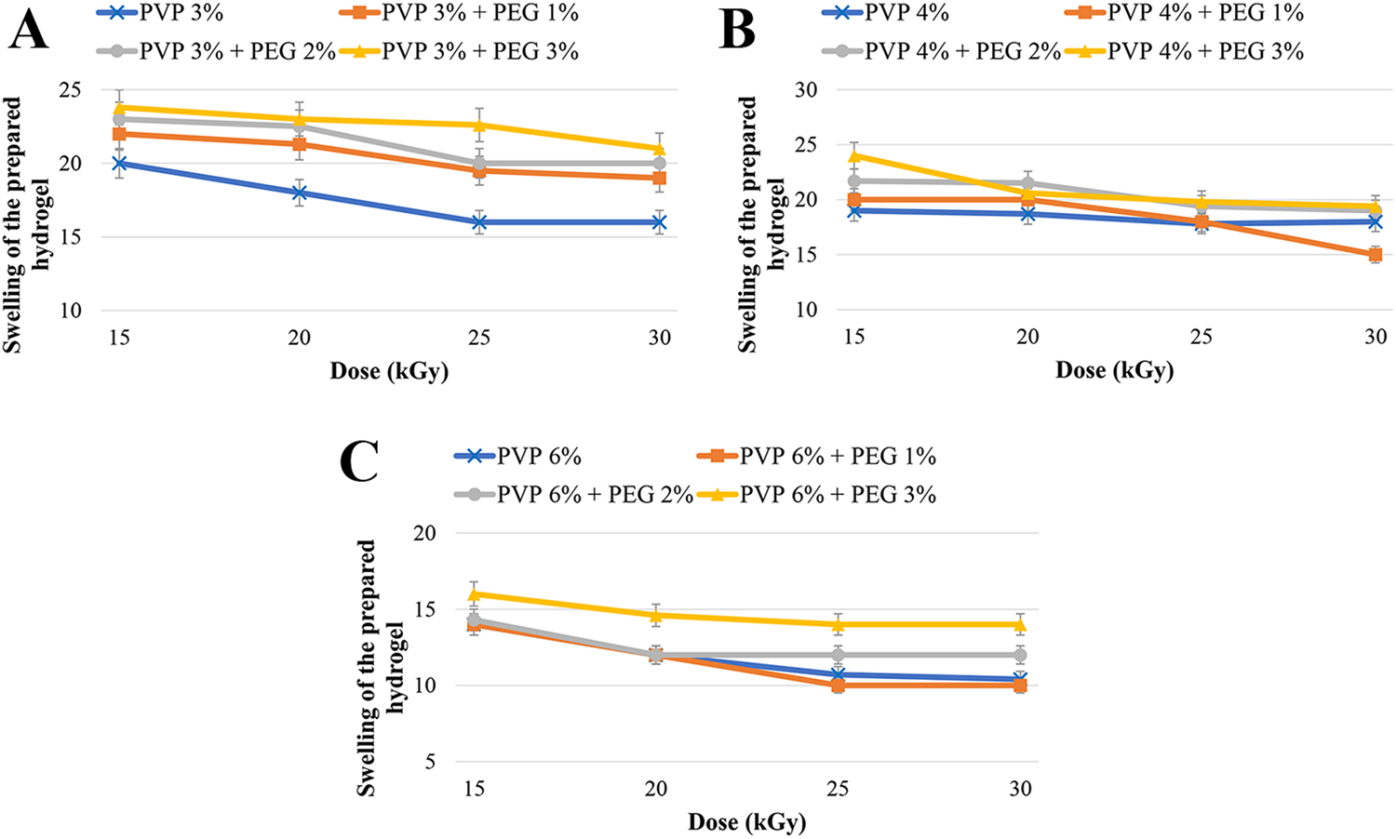
Effect of different radiation dose on the swelling degree in different formulations: (**A**) PVP 3% gels, (**B**) PVP 4% gels and (**C**) PVP 6% gels.

The results of swelling degree in the present study shown in Table 2 (Supplementary Information) and Fig. 2 indicated that the swelling was generally increased by increasing PEG concentration and decreasing radiation dose. In other words, the amounts of swelling degree were inversely proportional with cross-linking density. Although these rules were not definite in the present study, the prepared hydrogels were generally followed these principles which were consistent with the results of previous studies^15,23^. Also, the swelling degree in thiolated chitosan (19.8%) was less than chitosan (21%) hydrogels, indicating that TCH had higher extensibility and elastic modulus than chitosan hydrogel.

The most common technique used for prolonging the residence time of topical drugs at the application site is to enhance formulations viscosity using polymers. Therefore, in a polymeric network, rigidity and elasticity depend upon rheological properties^24^.

#### Evaluation of the hydrogels rheological properties

Rheological measurements afford a quantitative characterization of the visco-elastic characteristics of a substance under the defined conditions^25^. These measurements provide valuable information about the behavior and probable behavior of various products, in terms of their viscosity and elasticity^26^. Flow behavior is an indirect measurement of product consistency and quality and is associated with several parameters, such as molecular weight and its distribution.

**Figure 3:**
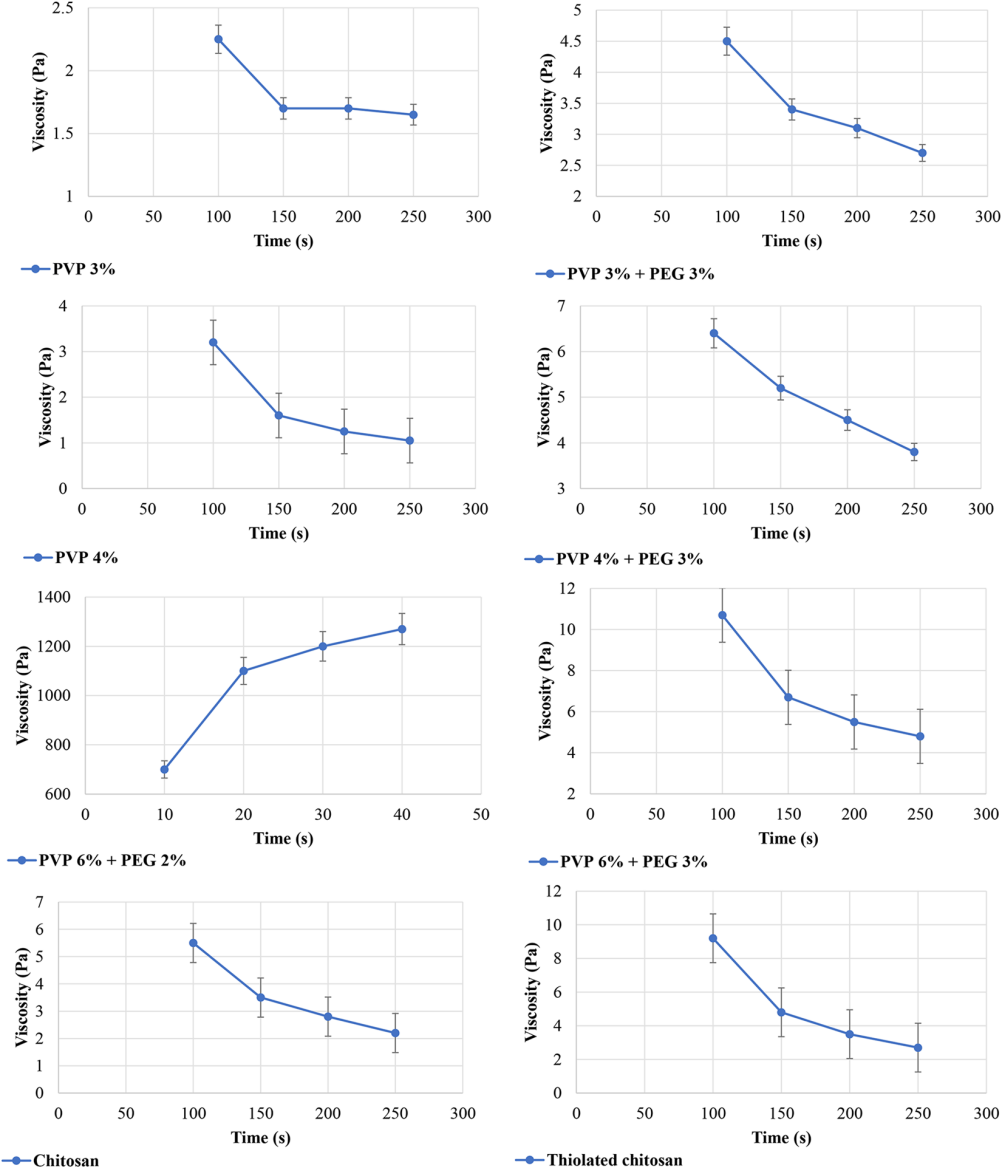
Flow properties of the various hydrogels.

**Figure 4:**
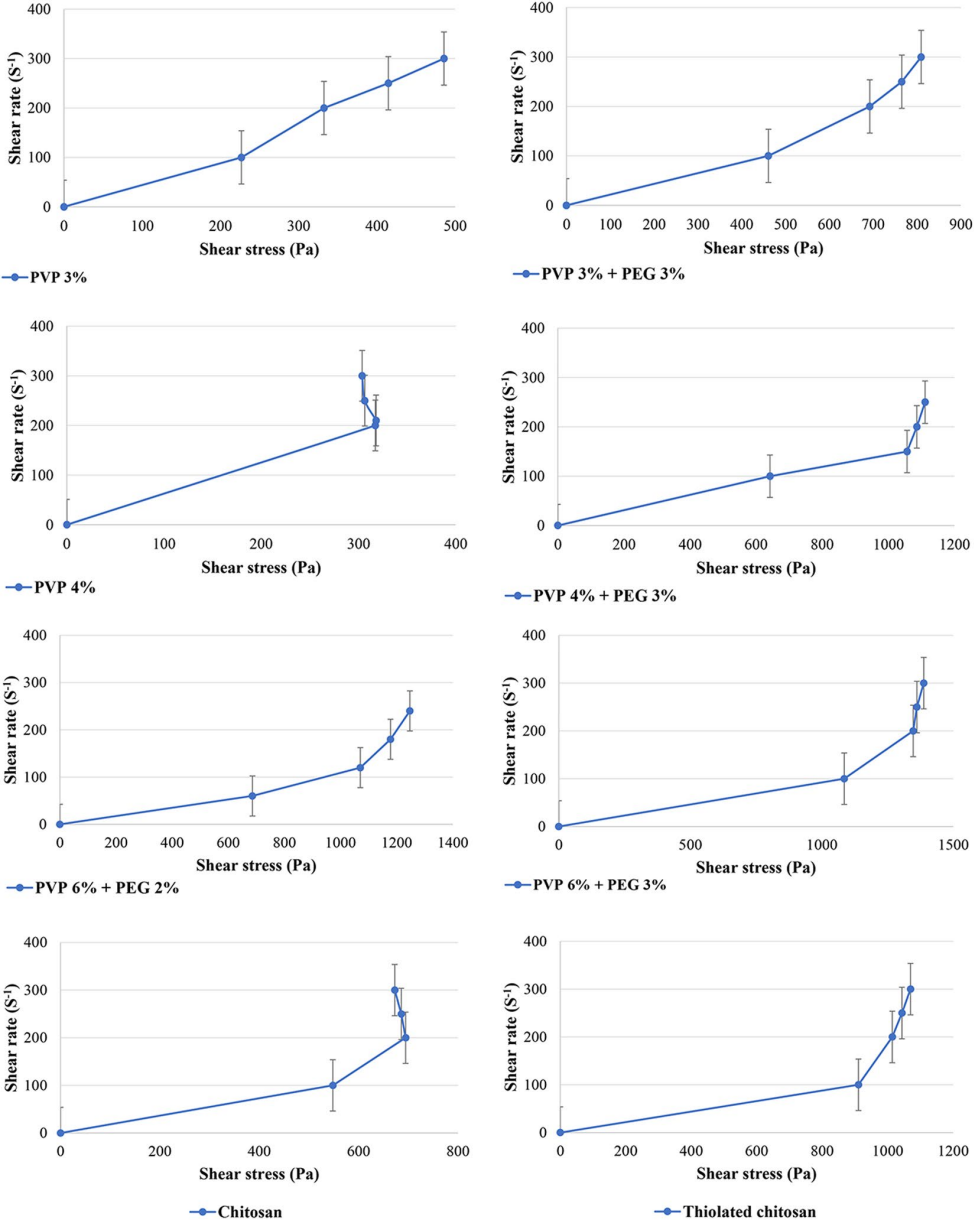
Flow curves of the various hydrogels.

In the current study, the results of rheological measurements indicated an inverse relationship between viscosity and shear rate, indicating shear thinning behavior of the samples (Figs 3 and 4). However, in the hydrogel prepared from PVP 6% and PEG 2%, there was an inverse phenomenon, in which the viscosity was increased by increasing the shear rate over time, known as rheopexy, as shown in Table 3 (Supplementary Information) and Fig. 3. Decreasing the viscosity of formulations under shear stress in a time-dependent manner is known as thixotropy. Also, the results showed that the shear thinning behavior was more obvious in those hydrogels prepared from PVP 4% compared to those prepared from PVP 6%. Moreover, TCH compared to chitosan showed less viscosity, and it was observed that TCH elasticity had a correlation with the polymer-linked thiol groups as shown in Table 3 (Supplementary Information) and Figs 3 and 4^27^. Therefore, TCH derivatives could be considered as appropriate excipients for the preparation of liquid and semiliquid formulations, which could be stable on the site of drug application, such as mucous membranes^24^.

#### Mucoadhesion measurement of hydrogels

Contact time has a key role in bioadhesion as a sufficient contact time provides adequate hydration and swelling of the polymer and enhances the interpenetration of moisture and the formation of non-covalent interaction, resulting in the promotion of mucoadhesion^28^. Among the different instrumental variables influencing the mucoadhesive properties of polymers, contact force and time, and the removal speed of the probe from the mucosal tissue are the most efficient factors. Chain flexibility is the most important physical mechanism of mucoadhesion, where flexible polymer chains intend to interpenetrate between polymer chains and mucus to a suitable depth for developing a robust adhesive bond^29^. Also, diffusion theory explains the interpenetration of polymer and mucin (a glycoprotein in epithelial lining of respiratory tract) chains to an adequate depth to develop a semi-permanent adhesive bond. Adhesion force is enhanced with increasing the penetration degree of the polymeric chains^25^. This penetration rate is determined by the factors of diffusion coefficient, mobility, flexibility and the essence of the mucoadhesive chains and contact time^7^. According to the previous finding^30^, the interpenetration depth needed to develop an efficient bioadhesive bond was in the range of 0.2–0.5 μm.

**Figure 5:**
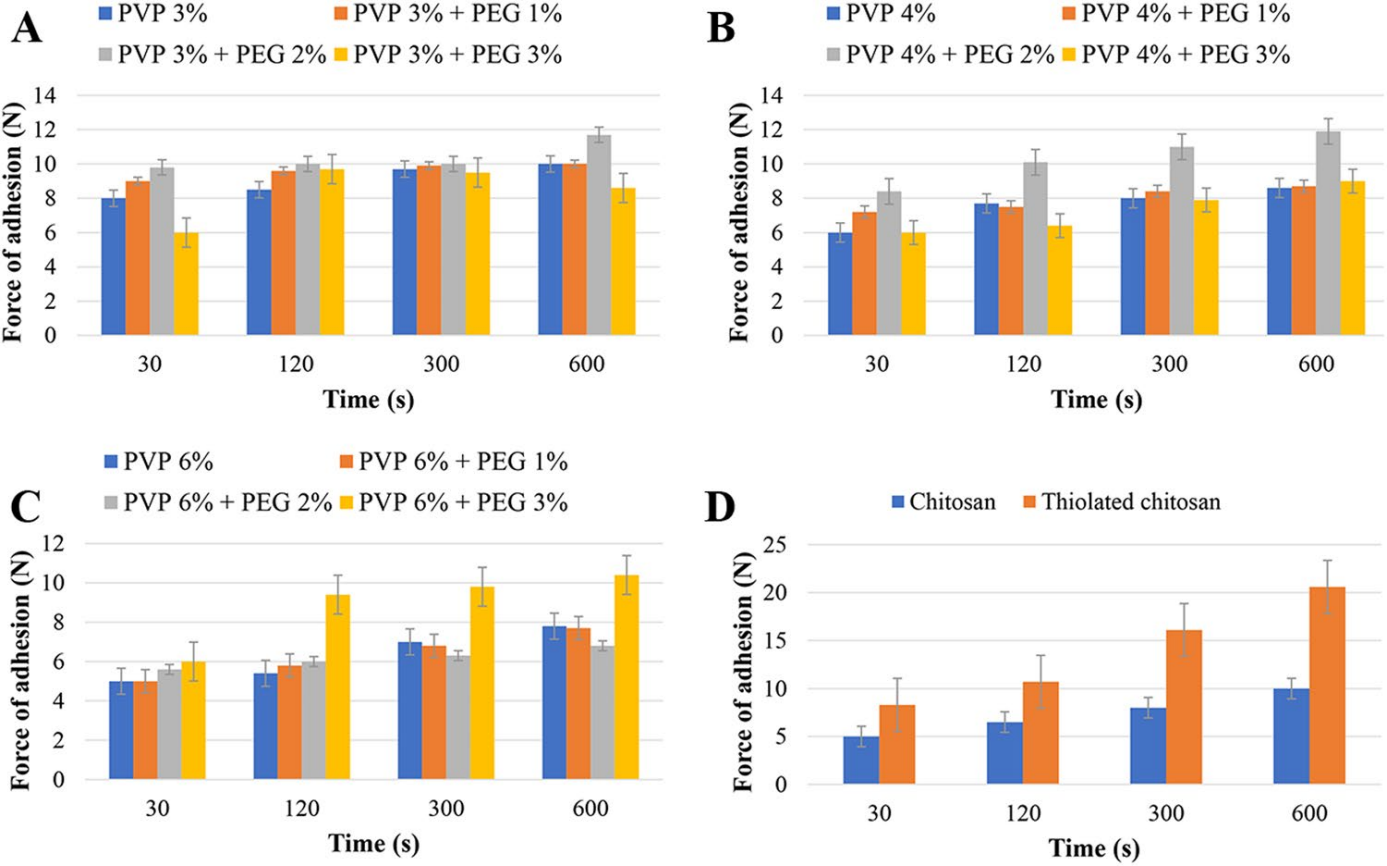
Effects of contact time on the adhesion force of different formulations: (**A**) PVP 3% gels; (**B**) PVP 4% gels; (**C**) PVP 6% gels; and (**D**) chitosan and thiolated chitosan gels.

In the present study, Tables 4 and 5 (Supplementary Information) and Fig. 5 illustrate the impact of contact time on the detachment force. It was found that the detachment force was increased with increasing the contact time. This was in consistent with the results obtained by Wong *et al*.^31^. The results, also, showed that TCH and the hydrogel prepared from PVP 6% and PEG 2% had the highest and lowest mucoadhesive intensity, respectively. Although the hydrogel prepared from PVP 4% and PEG 2% showed the highest force of mucoadhesion among PVP hydrogels, it had poor flexibility, while PVP 4% and PEG 3% hydrogel had less mucoadhesive properties but with better flexibility (Tables 4 and 5 (Supplementary Information), Fig. 5). Furthermore, it was found that, in all formulations, detachment force was decreased by increasing PVP concentration, while the force was increased with increasing PEG concentration. This finding was related to the PVP concentration as increasing PVP concentration causes the hydrogels to become more rigid, while PEG increases the elasticity and the detachment force. Cationic polymers, such as chitosan, have a good bioadhesive property and swelling in contact with nasal mucosa^32^. TCH constitutes disulfide bonds with mucus glycoproteins through reduced thiol groups, which are available on the chitosan backbone. The presence of disulfide bonds inter- and intra- molecularly makes chitosan as a highly stable drug carrier^30^. The formation of disulphide bonds between TCH and cysteine rich sub domains of mucus glycoproteins will augment the mucoadhesiveness properties of TCH^33^. The chitosan cross-linking depends upon the accessibility of the cationic sites and the negatively charged species^34^.

### *In vitro* and *ex-vivo* drug release from hydrogels

Drug delivery systems with controlled release properties are a new approach for the treatment of different diseases. In these systems, interactions between the drug, polymers and the used medium are the key factors which determine the release rate magnitude and release order^35^. The ability of a drug delivery system to extend the drug release in a controlled manner is defined as controlled release. In this regard, brain diseases are specially good candidate for controlled release methods, due to the presence of BBB^36^.

**Figure 6:**
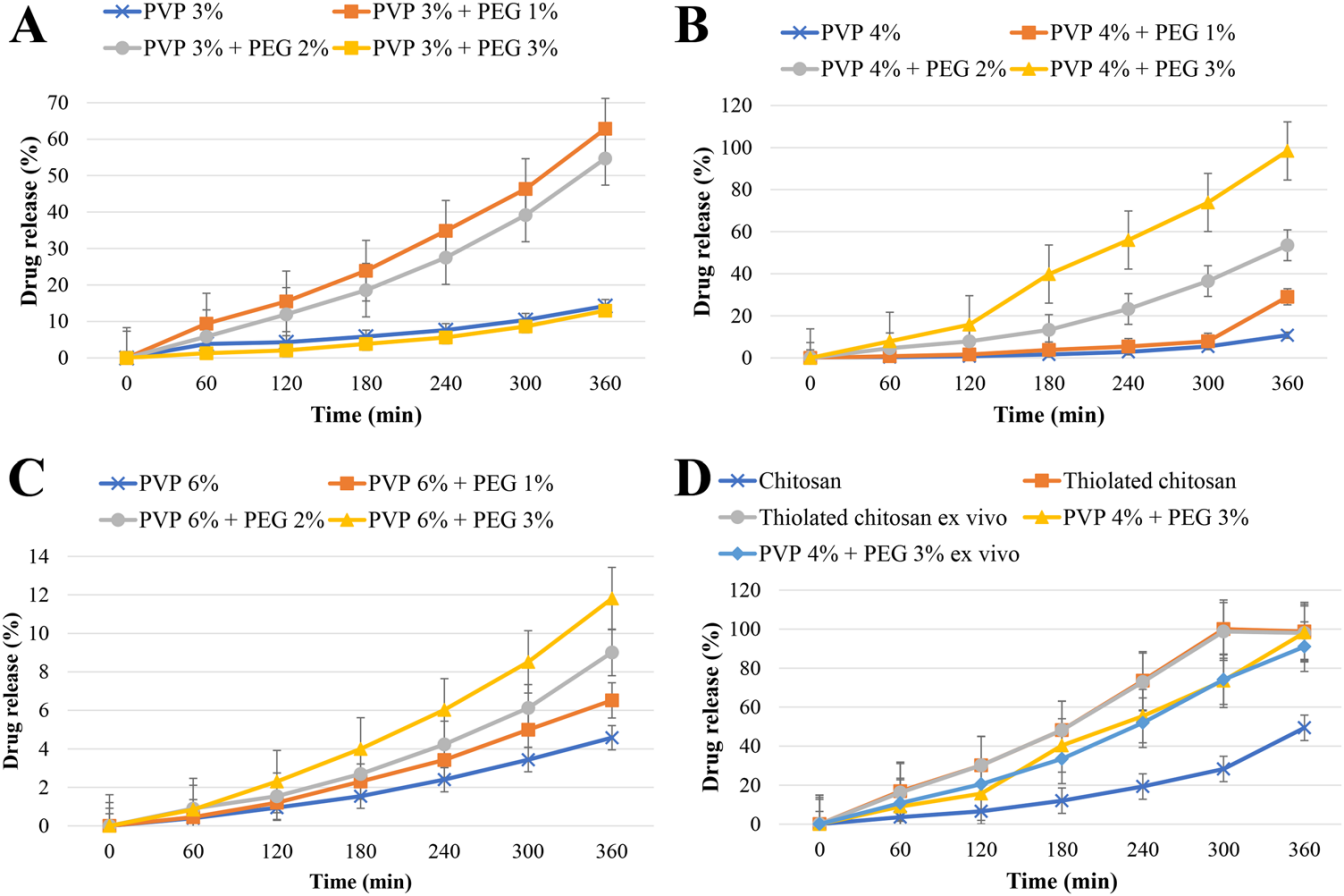
DH release profile from different formulations: (**A**) PVP 3% gels; (**B**) PVP 4% gels; (**C**) PVP 6% gels; and (**D**) chitosan, thiolated chitosan, thiolated chitosan *ex vivo*, PVP 4% + PEG 3%, and PVP 4% + PEG 3% *ex vivo*.

Figure 6 and Table 1 show DH release from different hydrogels formulations. The formulation contains PVP 4% and PEG 3% hydrogel showed the highest release rate (98.25%) of the drug among PVP hydrogels. These hydrogels were prepared by cross-linking at the radiation dose of 20 KGy as the efficient dose. It was identified that increasing the PVP content results in a decrease in the drug release rate, owning to the enhancement of the cross-linking density. In addition, with the increase of PVP concentration, the network formation is enhanced, and the elasticity and flexibility are decreased (Fig. 6). In contrast, PEG incorporation into hydrogels reduces the cross-linking density, network formation, and consequently increases the elasticity and flexibility, resulting in the enhanced release rate of drug. PEG is a hygroscopic molecule and absorbs water molecules from the release medium, leading to increasing the dissolving medium for the drug, reducing the hydrogel viscosity and increasing the drug release. By increasing the polymer concentration, the viscosity of hydrogel also increases, resulting in reducing the drug release. Ko *et al*.^37^ study showed the relationship between the profile of drug release and viscosity of chitosan solution. Also, the current study showed that TCH had higher drug release rate compared to chitosan and hydrogels prepared from PVP 3% and PEG 3%. This is due to the fact that chitosan functions as permeation enhancer, and this effect intensifies in the presence of thiol groups^38^. Moreover, chitosan can increase the paracellular absorption route, which has a key role in the transmission of hydrophilic therapeutics across the membrane^32^. Table 1 demonstrates the accumulative release profiles of DH from hydrogels. The formulations (PVP 6%, PVP 6% and PEG 3% hydrogels) seem to be fit with Korsmeyer-Peppas mathematical model (r > 0.999). These findings were in agreements with previous studies’ results^39,40^. In conclusion, these findings indicated that the prepared hydrogels had the controlled drug release pattern, making them suitable for biomedical applications.

**Table 1:** Release rate constants and correlation coefficients (r) obtained after fitting various mathematical models into the release profile of DH from hydrogels formulations.

Gel type	Zero-order^83^ Q_t_ = Q_0_ + K_0_t		Higuchi model^83^ F_t_ = Q = K_H_ × t^1/2^		First-order^83^ LogC = logC_0_ − Kt/2.303		Korsmeyer-Peppas^83^ Mt/M = Kt^n^			Hixson-Crowell^83^ W0^1/3^ − Wt^1/3^ = kt	
	K	r^2^	K	r^2^	K	r^2^	N	K	r^2^	K	r^2^
PVP 3%	0.036	0.9830	0.550	0.9323	0.00	0.9811	0.92	0.006	0.9853	0.000	0.9817
PVP 3% + PEG 1%	0.30	0.9642	0.444	0.8763	0.000	0.9603	0.91	0.008	0.9960	0.00	0.9616
PVP 3% + PEG 2%	0.133	0.9836	5.828	0.9571	0.002	0.9674	0.90	0.056	0.9973	0.001	0.9729
PVP 3% + PEG 3%	0.158	0.9903	2.422	0.9271	0.002	0.9735	0.9	0.094	0.9970	0.001	0.9795
PVP 4%	0.021	0.8990	0.302	0.7858	0.000	0.8948	0.97	0.001	0.9952	0.00	0.8962
PVP 4% + PEG 1%	0.046	0.8052	0.663	0.6894	0.000	0.7938	0.99	0.000	0.9878	0.000	0.8962
PVP 4% + PEG 2%	0.122	0.9646	1.831	0.8739	0.001	0.9444	0.98	0.025	0.9979	0.000	0.9511
PVP 4% + PEG 3%	0.246	0.9883	3.729	0.9144	0.004	0.9508	0.97	0.115	0.9956	0.001	0.9636
PVP 6%	0.011	0.9867	0.171	0.9110	0.000	0.9857	0.96	0.002	0.9994	0.000	0.9861
PVP 6% + PEG 1%	0.009	0.8357	0.142	0.8523	0.000	0.8468	0.92	0.007	0.8605	0.000	0.8464
PVP 6% + PEG 2%	0.021	0.9735	0.314	0.8891	0.000	0.9713	0.95	0.003	0.9976	0.000	0.9717
PVP 6% + PEG 3%	0.028	0.9839	0.427	0.9041	0.000	0.9812	0.96	0.002	0.9993	0.001	0.9821
Chitosan	0.102	0.9445	1.519	0.8482	0.001	0.9263	0.92	0.007	0.9926	0.000	0.9323
Thiolated chitosan	0.323	0.9923	4.943	0.9265	0.010	0.9320	0.95	0.132	0.9991	0.002	0.9498

### Histopathological effects of the hydrogels

Drug delivery systems must be safe for human clinical use^41^. In this regard, histopathological studies are commonly used for the toxicity assessment of formulations. Olfactory mucosa in sheep similar to other mammalians, such as human, constitutes of the olfactory epithelium and basal lamina propria. The olfactory epithelium by itself constitutes of basal, receptor and supporting cells, while Bowman’s glands in lamina propria are limited to the superficial part of the propria^42^.

**Figure 7:**
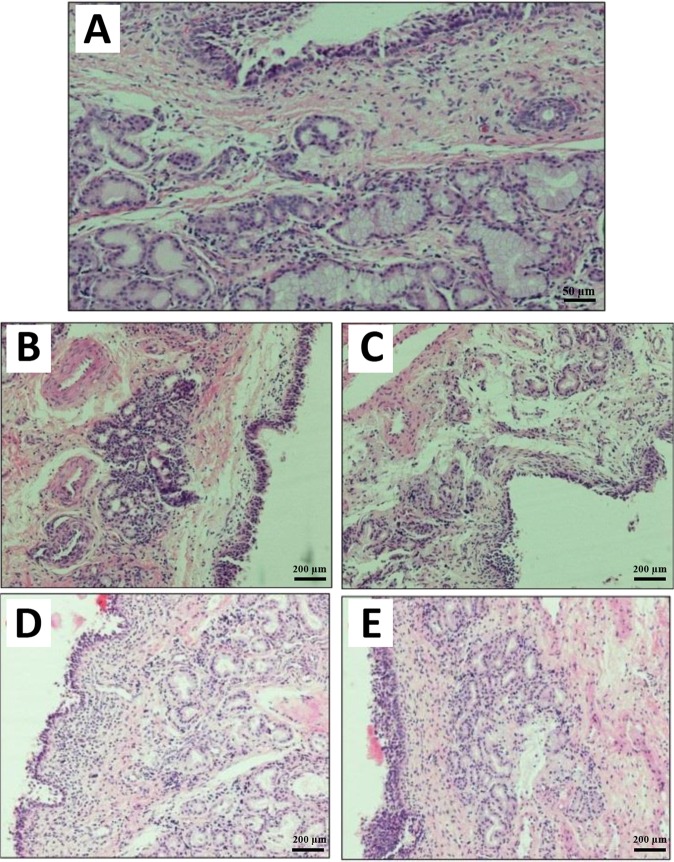
Histopathological photomicrograph of (**A**) the normal sheep nasal mucosa (control) (magnification size × 20); (**B**) nasal mucosa after treatment with hydrogels without additives. Hypotrophy in the Bowman’s gland and cellular infiltration in the connective tissue lamina propria surrounding the gland are perceivable; (**C**) nasal mucosa after treatment with the PVP + PEG hydrogels; (**D**) histological characterization of nasal mucosa after using chitosan hydrogel; and (**E**) nasal mucosa after treatment with thiolated chitosan hydrogel (magnification size × 10).

The findings of the present study showed that PVP hydrogels without additives led to a slight histological change in the olfactory mucosa (Fig. 7). There was hypotrophy in the Bowman’s gland and cellular infiltration in the connective tissue lamina propria surrounding the gland compared to the control group, normal respiratory epithelium with preserved cilia. The olfactory epithelium appeared to be not affected. Hydrogel containing PVP 4% and PEG 3% led to substantial histological changes in the mucosa of the olfactory epithelium, in which degeneration and sloughing of the epithelium surface were observed with ulcers in some regions of the epithelium. Degeneration and cellular infiltrations in the Bowman’s gland, dilation and congestion of the blood vessels were also observed. Other changes were apoptotic nuclei in the connective tissue lamina propria and Bowman’s gland. In the case of chitosan hydrogel (Fig. 7C), a marked cellular infiltration in the connective tissue lamina propria, apoptosis, and sloughing of the epithelium surface were observed associated with stratification of the epithelium (hyperplasia) in some regions. However, TCH (Fig. 7B) did not cause significant histological changes, in which the olfactory mucosa seemed to be similar to that of the control group with minor histological alterations. In this case, few degenerative changes in the epithelium surface and in the Bowman’s gland in some regions of the olfactory mucosa were observed. The ability of mucoadhesive polymers to interact with the mucus layer makes them suitable for incorporating with liposomes to inhibit the liposome clearance and increase the absorption of drugs loaded into liposomes^43^.

### Characterization of liposomes

Liposome is a small vesicle with the composition identical to the cell membrane^44^. Reverse-rotary evaporation, as a simple operation, is used for producing high envelopment monolayer liposome^45^.

**Figure 8:**
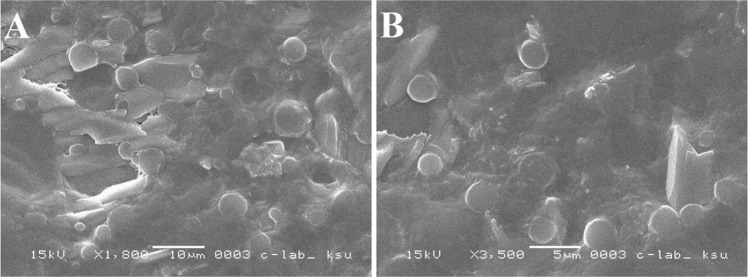
(**A**) SEM image of liposomes prepared by first reversible evaporator phase method; (**B**) SEM image of liposomes prepared by second reversible phase method.

In the present investigation, liposomes were prepared by two methods, and both were based on the reverse phase evaporation technique. In the first method, a short time sonication was used to prepare a water/oil emulsion from a two-phase system containing phospholipids in chloroform and phosphate buffer saline (PBS) containing DH. The liposomes were formed by removing the residual solvent using continuous rotary evaporation under reduced pressure, while in the second method, the hydrogel was subjected to the strong vortex to convert the hydrogel into suspension. The liposomal size influences the drug encapsulation efficiency in liposomes^46^. The results of various studies showed that using reverse phase evaporation technique led to vesicles with the mean diameter of 200–500 nm^47,48^. The vesicles size prepared by this method relies on the lipid composition and solvent. In this study, the size of the prepared liposomes was 438.7 ± 28.3 nm. Also, the results of SEM imaging (Fig. 8) showed that the LDH particles were prepared as large unilamellar vesicles with both oval and spherical shapes. In addition, the drug entrapment efficiency of prepared liposomes using the first and second methods was found to be 38% ± 0.7 and 62.5% ± 0.64, respectively. The second method is widely used to prepare liposomes since this method helps to increase drug encapsulation efficiency to 77–79%^49^.

### *In vitro* drug release from LDH incorporated into TCH

DH is freely soluble in water and can produce effective complexation, due to the desired ionization power^50^. The factors of hydrogel composition (type of polymer, drug and additives), geometry (size and shape)^51^, preparation method (*in-situ* drug loading and post loading methods)^52^, and environmental conditions during drug release determine the mechanism of release^51^. Drug loading method and physicochemical properties of the lipid membrane are the most important factors, influencing drug release rate from liposomes^53^.

**Figure 9:**
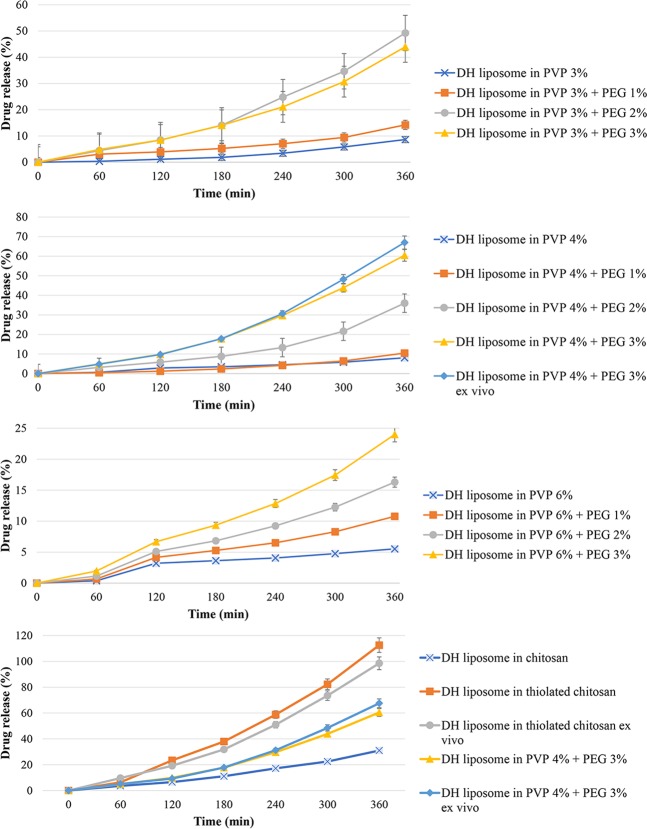
The profile of DH release from various liposomal hydrogels.

In the current study, the drug release from LDH incorporated into TCH was less than LDH as positive control (Fig. 9). The higher release rate of DH from liposomes compared to liposomes incorporated into TCH might attribute to the retarded release from hydrogel^54^. As Fig. 9 shows, drug release from liposomes incorporated into TCH was higher (94.7%) than the most hydrogels, which might be resulted from the polymer linkage. This suggested some defects occurred in the bilayer liposomes, due to applied forces on the phospholipid head-groups by intermolecular crosslinking^55^. Therefore, interactions between thiolated polymer matrix and LDH do not affect the controlled and sustained release profile^55^. The release profile of DH from liposome incorporated into TCH revealed best fit with Korsmeyer-Peppas model (Table 2)^56^.

**Table 2:** Release rate constants (K) and correlation coefficients (r) obtained after fitting various mathematical models into the release profile of LDH incorporated into TCH.

Mathematical models	Zero-order F = K_0_*t		Higuchi model *F* = KH*t ^*ʌ*^*0.5*		First-order F = 100* [1− Exp (−K1* t)]		Korsmeyer-Peppas F = kKP *t^ʌ^n		Hixson-Crowell F = 100*[1 − (1 − kHC*t)^ʌ^3]	
Hydrogel type	K	r	K	r	K	r	K	r	K	r
Thiolated chitosan	0.322	0.9917	4.931	0.9259	0.008	0.9306	0.152	0.9917	0.9998	0.9988

### Stability study

Stability is one of the important factors in the design and development of formulations. The assessment of size distribution is important to study the physical or colloidal stability of a formulation under storage conditions and in a biological medium^57^. The physical stability of liposomes could be studied by measuring the size of liposomes which is increased, owing to their aggregation and or fusion. The physical stability of liposomes can be increased by enhancing the amount of liposomes charge (positive or negative)^58^. Also, it has been found that the surface charge density of liposomes influences their distribution in an *in vivo* environment^59^. Drug release from liposome and change in liposome size could occur, due to those physical processes affecting the shelf life of liposomes, such as aggregation/flocculation and fusion/coalescence. Often a colloidal dispersion is thermodynamically unstable^60^. Liposomal formulations which are physically stable preserve size distribution and drug entrapment efficiency. Such stability is determined by different factors, such as thermodynamics and colloidal properties of the system^54^. The size of particles for nasal delivery should be above 10 µm because the smaller particles (<10 µm) can be transferred to the tracheobronchial with the airstream, while larger particles will mostly deposit in the nose. Therefore, the size range of 40–60 µm is appropriate for nasal delivery^61^. The size of particles prepared in the present study ranged from 45–58 µm.

**Figure 10:**
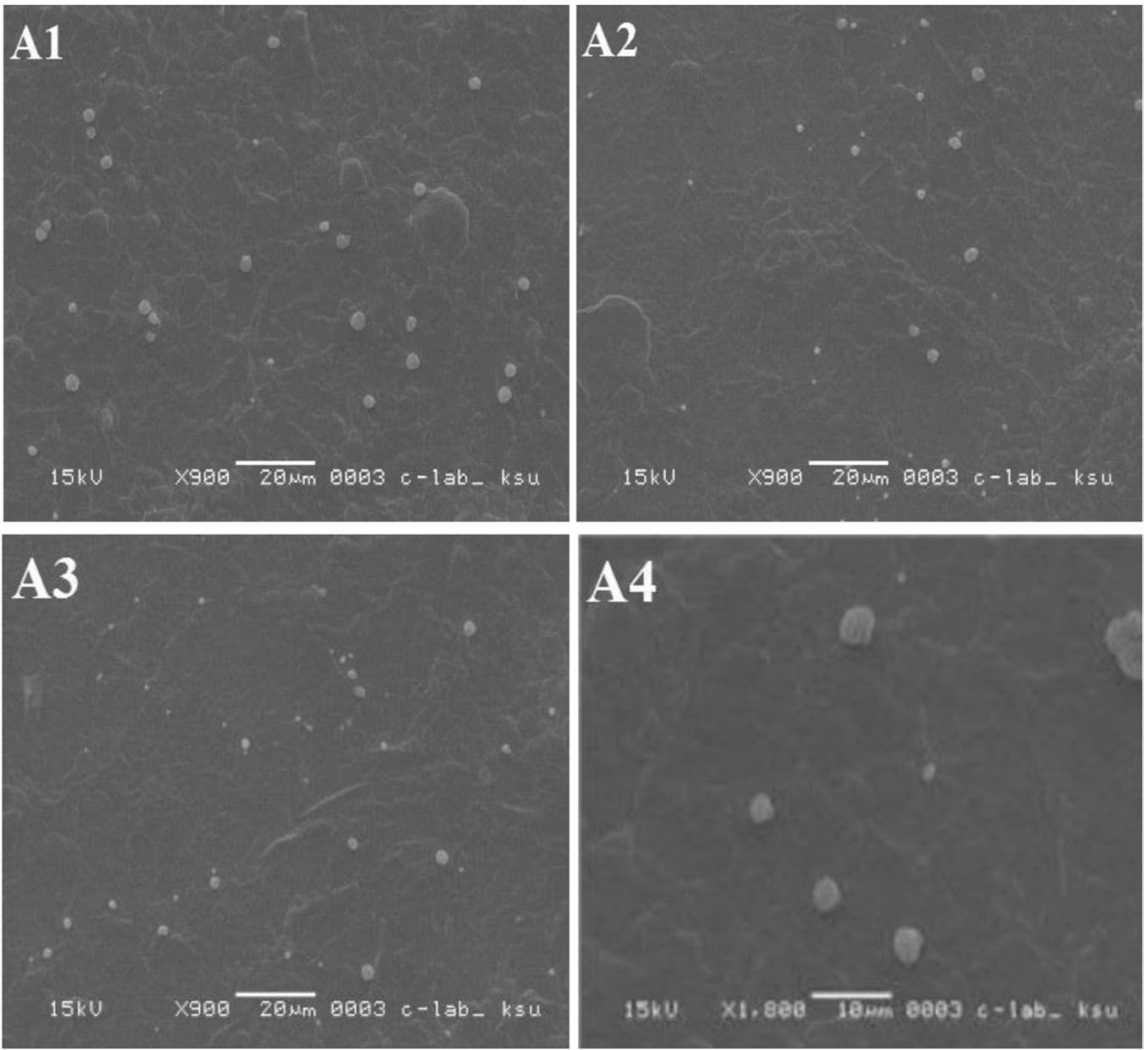
SEM images of LDH incorporated into TCH during stability study, (**A1**) liposomes stored at 4 °C for one week; (**A2**) liposomes stored at 4 °C for 2 weeks; (**A3**) liposomes stored at 4 °C for 3 weeks; and (**A4**) liposomes stored at 4 °C for 4 weeks.

**Figure 11:**
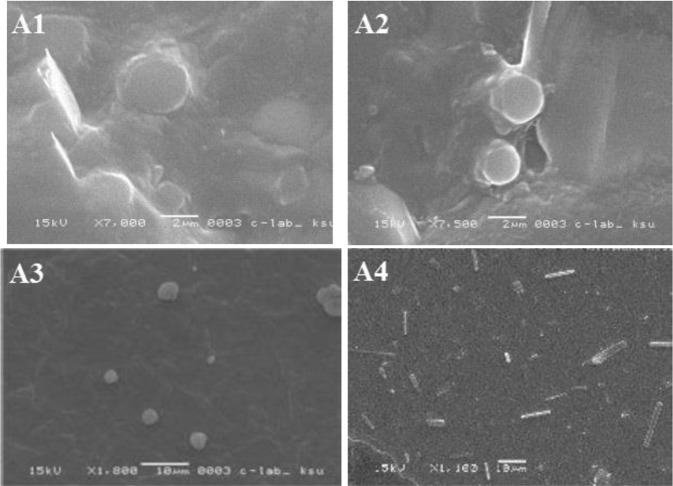
SEM images of LDH incorporated into TCH during stability study, (**A1**) liposomes stored at 20 °C for one week; (**A2**) liposomes stored at 20 °C for 2 weeks; (**A3**) liposomes stored at 20 °C for 3 weeks; and (**A4**) liposomes stored at 20 °C for 4 weeks.

**Figure 12:**
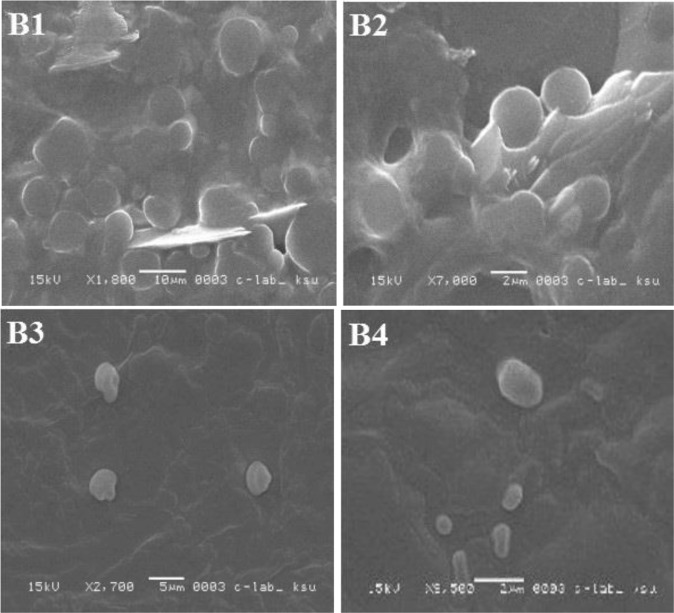
SEM images of LDH during stability study, (**B1**) liposomes stored at 4 °C for one week; (**B2**) liposomes stored at 4 °C for 2 weeks; (**B3**) liposomes stored at 4 °C for 3 weeks; and (**B4**) liposomes stored at 4 °C for 4 weeks.

**Figure 13:**
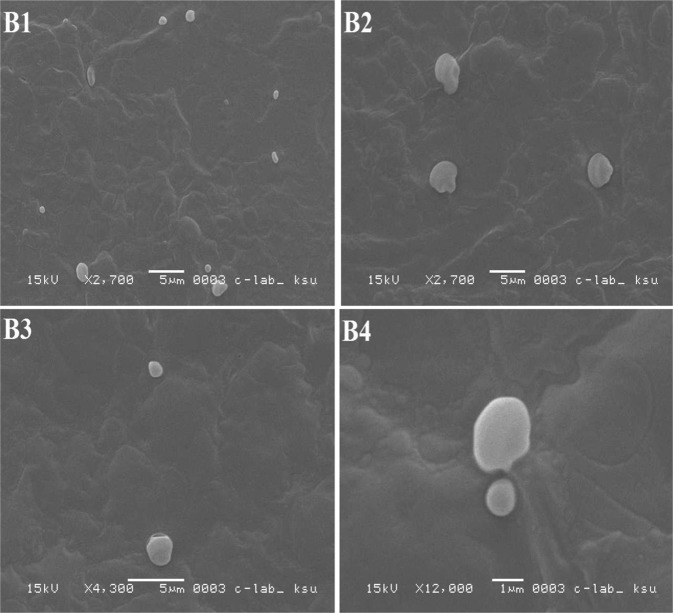
SEM images of LDH during stability study, (**B1**) liposomes stored at 20 °C for one week; (**B2**) liposomes stored at 4 °C for 2 weeks; (**B3**) liposomes stored at 20 °C for 3 weeks; and (**B4**) liposomes stored at 20 °C for 4 weeks.

The storage results of the current study for various formulations were indicated in Figs 10–13. It was found that the morphology of liposome was affected by the temperature, where it was changed remarkably at 20 °C, while it was approximately stable at 4 °C. It was perceived that liposomes incorporated into TCH were considerably more stable than the unincorporated ones (Fig. 13).

### Chromatographic system and conditions

**Figure 14:**
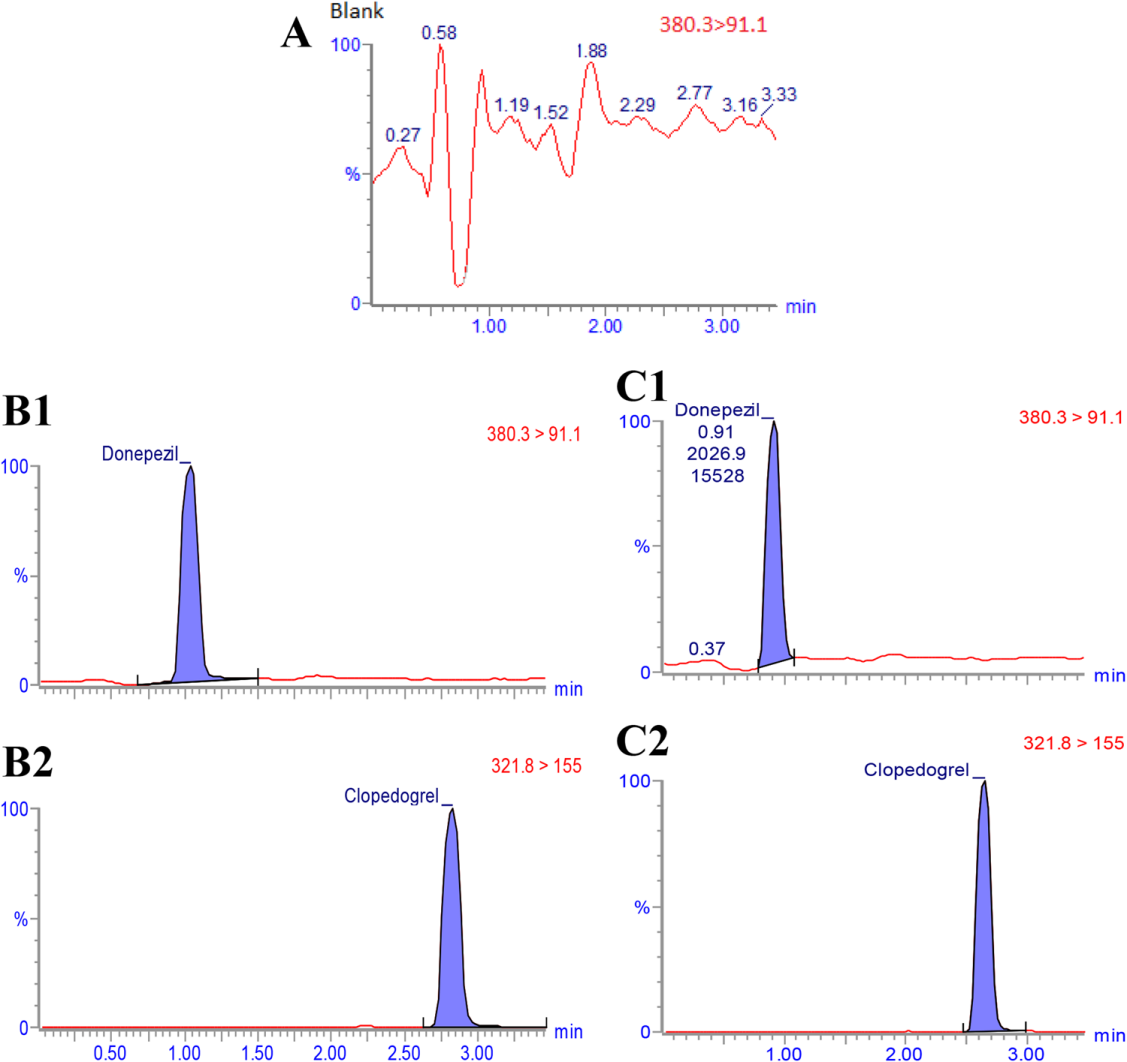
(**A**) Chromatogram of blank plasma; (**B**) UPLC MS/MS chromatogram of extracted of spiked plasma with 1 ng DH and 40 ng IS; (**C**) UPLC MS/MS chromatogram of the extracted of DH 12 h after intranasal administration to rabbits and multiple reaction-monitoring (MRM) transitions of IS.

In the present study, the blank plasma (Fig. 14A) in comparison to the spiked plasma samples did not show significant interfering endogenous peaks at the retention times corresponded to DH (Fig. 14B1,C1) and IS (Fig. 14B2,C2), indicating the specificity of the assay. The chromatographic behavior of QC samples was similar to that of actual plasma samples (Fig. 14B1 vs. B2,C1 vs. C2).

The chromatograms resulted from plasma sample containing DH and IS collected 12 h after nasal administration of LDH incorporated into TCH to the rabbits are shown in Fig. 14C. The peaks corresponded to DH and IS were distinct with the retention times of approximately 1 min and 2.6 min, respectively. The chromatographic run time of 3.5 min was sufficient for sample analysis.

To evaluate the linearity of the method, calibration curves were constructed using the six series of plasma samples containing 1–50 ng/mL of DH. The linear relationship (Y = 0.013 + 0.251X) was obtained between the peak area ratio of DH to that of IS versus the corresponding concentration of DH (with correlation coefficient (r) of >0.992 for all tested standard calibration curves). The validation of the calibration curve linearity was confirmed using high value of the correlation coefficient. The precision values for intra- and inter-day were <16.5% and the accuracy was <17.6 for DH concentrations^62^.

### Pharmacokinetic study

After validating of the proposed bio-analytical method, it was successfully used to measure the plasma DH concentration for pharmacokinetic study in the New Zealand white rabbits. Aside from the economic reasons, rabbits are common laboratory animals because of their size, temperature and ability to produce genetically homogeneous strains^63^. In the current study, New Zealand white rabbits, as a well-controlled animal model, were used to evaluate the absorption potency of formulations from nasal route^64^.

**Figure 15:**
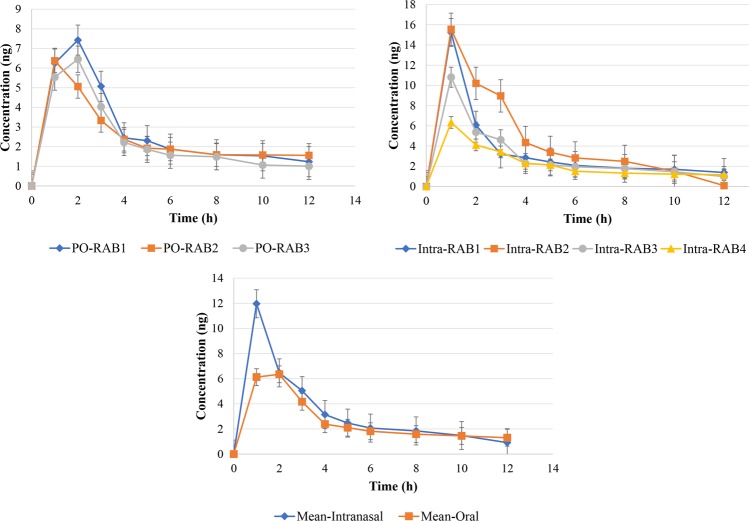
DH plasma concentration time profiles in rabbits after intranasal administration of LDH incorporated into TCH (n = 6) and oral administration of oral DH tablet (n = 6).

Time profiles of DH plasma concentration (mean ± SD) in the rabbits after oral administrations of 5 mg of DH tablet and 5 mg of nasal LDH incorporated into TCH were shown in Fig. 15. The pharmacokinetics parameters for the tablet and LDH incorporated into TCH after oral and intranasal administration of DH are summarized in Table 3. It was found that the maximum plasma concentration of DH was achieved 1 h after the intranasal administration of LDH incorporated into TCH and 1.7 h after oral tablets administration. C_max_ was 12 ± 1.3 ng/mL and 8.2 ± 1.4 ng/mL for intranasal administration of LDH incorporated into TCH and oral administration of DH tablets, respectively, indicating the potency of liposome incorporated into TCH to increase C_max_ through nasal route by 46%. Moreover, intranasal formulation increased the AUC by 39% compared to the oral tablets of DH. Overall, the results of DH measurement in the blood and brain proved that the intranasal DH delivery through LDH incorporated into TCH caused higher DH concentration in the blood circulation and brain compared to the oral tablets of DH. The results showed that the mean brain content of DH was >2-fold through intranasal administration of LDH incorporated into TCH compared to the oral tablets. This confirmed that the intranasal LDH incorporated into TCH is superior for DH delivery to the rabbit brain compared to the oral tablets.

**Table 3:** Pharmacokinetics parameters for the tablet and intranasal LDH incorporated into TCH in rabbit plasma and brain content.

Rabbit number	Intranasal					Tablet			
Properties	1	2	3	4	Mean ± SD	1	2	3	Mean ± SD
Dose (mg)	0.4	0.4	0.4	0.4	0.4 ± 0.0	0.4	0.4	0.4	0.4 ± 0.0
AUC_(0-t)_, (mg/l)h	15.1	33.4	16.3	12.3	19.3 ± 2.1	16.4	11.4	13.9	13.9 ± 2.5
C_max_, (ng/ml)	15.2	15.4	10.9	6.3	12.0 ± 1.3	7.4	6.4	10.9	8.2 ± 1.4
T_max_, (h)	1.0	1.0	1.0	1.0	1.0 ± 0.0	2.0	1.0	2.0	1.7 ± 0.2
T_1/2_, (h)	8.4	1.1	7.4	6.2	5.8 ± 0.7	8.4	9.5	5.3	7.7 ± 2.2
MRT, (h)	17.5	5.4	14.2	14.7	13.0 ± 1.4	15.9	20.7	12.6	16.4 ± 1.5
CI/F, (L/h)/kg	3.1	3	3.7	4.5	3.6 ± 0.4	3.2	3.1	4.6	3.6 ± 0.7
Brain content, (ng/g)	29.1	52.8	34.4	33.5	37.5 ± 3.5	4.7	16.6	33.1	18.1 ± 1.8

^*^MRT: mean residence time.

## Conclusion

In the current study, the synthesis of different DH hydrogels was reported for intranasal DH delivery. They were synthesized using gamma radiation and different natural and synthetic polymers. After *in vitro* characterization, TCH was selected for incorporating with LDH and subsequent pharmacokinetic evaluation in rabbits. The formulation was administered through nasal route. The *in vivo* results showed that LDH incorporated into TCH significantly increased the blood concentration and the brain content of DH compared to the oral tablets of DH. In conclusion, this study suggests further investigation of LDH incorporated into TCH in the animal model of Alzheimer disease.

## Materials and Methods

### Materials

DH was provided from Jai Radhe Sales (Gujarat, India). Chitosan (103.2 g/mol, degree of deacetylation 76.6%), 2-imino thiolane hydrochloride, 2-mercaptoethanol, triethanolamin reagent grade, formic acid (ultra-performance liquid chromatography MS/MS (UPLC MS/MS) grade), clopidogrel (as internal standard (IS)), ammonium formate, and acetonitrile were purchased from Sigma Aldrich Company (St. Luis, MO, USA). Cholesterol (CHOL) and dipalmitoylphosphocholine (DPPC) were purchased from Fisher Scientific Company (Fair Lawn, NJ, USA) and Avanti Polar Lipid Inc., (Birmingham, AL, USA), respectively. Polyvinyl pyrolidone (PVP), known as Luviskol® K-9, was purchased from BASF (Aktien gesellschaft Ludwig Shafen, Germany). Polyethylene glycol 600 (PEG600) was obtained from Merck Schuchardt (Hohenbrunn, Germany). Lactic acid glutaraldehyde 50% solution and chloroform were purchased from BDHL laboratory Supplies (PooleBH151TD, England). Mono basic potassium dihydrogen phosphate and cellophane membrane (Spectra/Por® Dialysis Tubing from regenerated cellulose) with cut-off 12–14000 Da were purchased from panreac Quimica, SA, (Barcelona, Espana) and Spectrum Laboratories (Houston, TX), respectively. All chemicals used were of analytical grade and were used as received.

### *In vitro* study equipment

Drug diffusion from different formulations was evaluated using a vertical jacketed 15 mL Franz Cell with 12 mL receptor volume provided by PermeGear Inc. (3575 North Drive, Bethlehem, PA 18015, Ref n.: #6G-01-00-20-15). Rotavapor R-210 from Büchi Labortechnik AG, Flawil, Switzerland was used for liposome preparation. Morphology of the formulations was evaluated using scanning electron microscopy (SEM) (JSM-6060; JEOL, Tokyo, Japan). The mean particle size of the formulations was determined by dynamic light scattering (DLS) using 90 Plus particle size analyzer (Laser nanoparticle size analyzer model no. 90 Plus- software version 3.74, Brookhaven Instruments, Holtsville, New York, USA). Milli-Q-Plus system (Millipore systems®, France) was used for preparing de-ionized water.

### Preparation of donepezil HCl-loaded PVP hydrogels

PVP hydrogel was synthesized based on the method described by Abd El-Mohdy *et al*.^17^ with some modifications. Briefly, PVP (3, 4, 6% w/w) with (1, 2, 3% w/w) and without PEG were prepared in 100 mL of deionized water, and the samples were stirred (5 min, 30 RPM). The solutions were transferred in glass bottles, and nitrogen was bubbled in the solutions to remove oxygen. Next, the bottles were sealed and irradiated at room temperature by gamma radiation produced from Cobalt-60 source with the doses of 15, 20, 25, 30 KGy. The products were fully sterile permanent hydrogels. The radiation time was measured according to the radiation source dosing rate (51.7 Gy/min). DH was also homogeneously dispersed in the hydrogels after crosslinking at the final drug concentration of 20 mg/g. The pH value of the prepared hydrogels was adjusted at 6.4 ± 0.15 by triethanolamin. The blank hydrogels were prepared without adding the drug.

### Preparation of donepezil-loaded chitosan hydrogel

The chitosan hydrogel was prepared according to Chen *et al*.^65^ study. For this purpose, 2 g chitosan were added into acetic acid solution (100 mL, 0.1 M) and stirred (room temperature, 150 RPM) to achieve a homogeneous solution. Chitosan contains -NH_2_ amino group which can interact with glutaraldehyde as a cross-linking agent^66^. The cross-linking reaction between chitosan and glutaraldehyde was carried out at room temperature for 4 h. The pH value of hydrogel was adjusted to nasal (pH 5.5 ± 0.15). Also, the drug was loaded into hydrogel as mentioned before.

### Preparation of donepezil HCl-loaded thiolated chitosan hydrogel

TCH was prepared according to the method described by Andreas *et al*.^67^ with some minor modifications. Briefly, a chitosan solution (1 g/700 mL of 1% acetic acid) was prepared, and 0.2 g of 2-iminothiolane HCl (Trauts reagent) was added. pH was adjusted to nasal (pH 5.5 ± 0.15). The oxidation process during the coupling reaction was inhibited using 2-mercaptoethanol at the final concentration of 3% (v/v). After 24-h incubation (room temperature, 200 RPM), the mixture was dialyzed against 5 mM HCl, 5 mM HCl containing 1% NaCl twice, 5 mM HCl, and 1 mM HCl, respectively, and then stored at 4 °C. The drug was incorporated as before.

### Characterization of hydrogels

#### Drug loading efficiency

Drug loading efficiency was indirectly measured; for this purpose, the swollen hydrogels incubated with DH were separately removed from the drug solutions, washed with 10 mL of distilled water, and were then dried in an oven at 37 °C for 24 h. The drug concentration remained in the solution was measured spectrophotometrically at 230 nm. The drug loading efficiency was then estimated using formula (1):

1 $$Drug\,loading\,efficiency=\frac{{W}_{dg}}{{W}_{g}}$$

where W_dg_ is the mass of the drug loaded into hydrogel and W_g_ is the amount of initial drug.

#### Gel fraction

The dried gels were immersed in hot water for 24 h, and water was changed every 4 h with fresh one. The gel fraction was determined by measuring the initial weight of dry gel before extraction and the weight of dry gel after extraction^15^ using formula 2.

2 $$Gel\,fraction\,( \% )=\frac{{W}_{f}}{{W}_{o}}\times 100$$

where W_f_ and W_O_ are the weight of dry gel before and after extraction, respectively.

#### Degree of swelling

The degree of swelling is defined as the water absorption capacity of hydrogel. The weighed dried hydrogels were immersed in redistilled water and incubated for 24 h (room temperature) to reach the equilibrium state of swelling. The superficial water on the swollen gel was then removed using tissue paper, and immediately weighed. The degree of swelling was calculated using formula (3):

3 $$Degree\,of\,swelling\,( \% )=\frac{{W}_{s}-{W}_{d}}{{W}_{d}}\times 100$$

where W_s_ is the weight of the swollen gel and W_d_ is the weight of dry gel^68^.

#### Evaluation of the hydrogels rheological properties

The rheograms of the bioadhesive hydrogels were obtained at 37 °C using a rheometer equipped with a cone and plate measuring system. Samples were carefully put into the plate, in which formulation shearing did not occur and the parameters of shear rate, shear stress, and viscosity were monitored to investigate the flow profile, zero-rate viscosity, and thixotropy. The rheograms were obtained from at least three determinations^69^.

### Mucoadhesion measurement of hydrogels

Mucoadhesion strength of the hydrogels was measured to evaluate their adhesion capability onto the mucus membrane. Nasal mucosal tissue was excised from the noses of freshly slaughtered sheep and stored on ice during transport to the laboratory. The nasal mucosa was immediately separated from the underlying cartilage (the time of tissue excision and mucosa separation should not be more than 30 min) by blunt stripping using a pair of tweezers^70^. An instron machine with a 5 K newton (N) load cell and 1.2 cm diameter metallic cylindrical probe were used for the evaluation of mucoadhesion properties. Mucosa was attached to the lower and upper end of the instrument probe using cyanoacrylate glue, and 200 mg of hydrogel samples were then placed on the lower mucosa. Upper probe which hold the nasal mucosa was lowered onto the surface of the hydrogels until the contact was occurred with the initial force of 0.5 N. After a contact period of 120 s, the upper probe was withdrawn and moved vertically upward to separate two surfaces with a constant speed of 5 mm s^−1^ until failure occurred between the surfaces. The mucoadhesion studies were performed at room temperature and 50% relative humidity. The data were collected, and the calculations were carried out using Instron® Series IX automated material tester software, version 8.32.00. Each measurement was repeated at least 5 times^70^.

The effect of contact times of 30, 120, 300 and 600 s was evaluated on the mucoadhesion properties of the various hydrogels. The instron was adjusted with a probe speed of 5 mm s^−1^ and a contact force of 0.5 N. In addition, a sheep nasal mucosal membrane was used as a biological membrane^70^.

### *In vitro* drug release form hydrogels

*In vitro* release studies were performed at 37 °C using the Franz diffusion cell. Briefly, 0.5 g of medicated formula containing 5 mg of DH was packed into each of three cell donor chambers, in which no air bubbles were produced between the formulation and donor surface of the cellophane membrane. The receptor phase was filled with PBS (pH 5.5) and continuously stirred (100 RPM) during the experiment to ensure homogeneity. The samples were taken at the different time intervals (0.5, 1, 1.5, 2, 2.5, 3, 3.5, 4, 4.5 and 5 h) and analyzed spectrophotometrically at the wavelength of 230 nm. The cumulative percentage of drug release was determined and plotted against time. All *i*n *vitro* drug release studies were performed in triplicate^70,71^.

### *Ex-vivo* drug release from hydrogels

*Ex-vivo* experiments were performed using excised sheep mucosal tissues. The nasal mucosa was prepared as mentioned at the section of mucoadhesion measurement. Samples were taken and inserted into the Franz diffusion chambers where the apical side of the tissue was typically faced with the donor compartment. Franz diffusion cells (final volume of 12 mL) were used to measure the DH release from the hydrogel formulations. For this purpose, 5 mg of the hydrogel containing 0.5 mg of DH was placed on the upper side of the nasal mucosa. The donor half-cell was then placed on the top of the receptor half-cell and clamped. The donor and the receiver compartments containing PBS (pH 5.5) were kept in the intimate contact and the temperature was maintained at 37 °C^72^.

### Histopathological effects of hydrogels

The sheep’s nasal mucosa was prepared as mentioned before. The mucosal tissues were incubated in the various hydrogels for 5 h at 25 °C, and the tissues were fixed in 10% formalin solution for 48 h. Next, the tissues were embedded in paraffin wax. They were then sectioned (7 µm) and placed on glass slides. Later, they were stained with hematoxylin and eosin (H&E). The sections were evaluated using a light microscope connected to a digital camera to measure any changes. Mucosal tissues incubated in isotonic PBS (5 h) were considered as a control for comparison^70^.

### Preparation of liposomes

Liposomes were synthesized using reverse phase evaporation technique. For this purpose, DPPC: CHOL (1.6:1 molar ratio) were dissolved in chloroform, and then the organic solvent was removed using rotary evaporator to form a thin film^73^. Next, 5 mg of DH was dissolved in PBS (pH 5.5), and the resultant was added into the thin film^74^.

Also, LDH was prepared by a method described by Turner *et al*.^63^. Briefly, 150 mg of DPPC and 50 mg of CHOL (1.6:1 molar ratio) were dissolved in chloroform in a round flask. Next, 5 mg of DH was dissolved in PBS and injected into lipid solution. The round flask was then capped with a glass stopper and sonicated for 5 min. Next, the solvent was evaporated using rotatory evaporator at 45 °C under vacuum condition, and then the resultant was vigorously vortexed for 5 min. The resultant was again evaporated to make sure the organic solvent was completely removed. The cycles of 10-min drying and 10-min vortexing were repeated thrice^64^.

### Characterization of liposomes

The mean particle size and the morphology of the liposomes were determined using laser particle size analysis and SEM imaging, respectively^75^. Also, drug entrapment efficiency was evaluated using dialysis method. Briefly, the liposome suspension was transferred into a dialysis tube membrane (donor compartment). The dialysis bag was immersed into 200 mL of PBS (pH 5.5) and stirred (120 RPM, 4 h). Next, the drug concentration in receptor compartment was determined through the measurement of absorbance at 230 nm^71,76^. The drug entrapment efficiency (EE) was then calculated using formula (4).

4 $$EE( \% )=\frac{initial\,drug\,concentration\,-\,drug\,concentration\,in\,receptor\,compartment}{initial\,drug\,concentration}\times 100$$

### Preparation of liposomal hydrogel

The incorporation of the liposomes into the TCH was achieved by slow mechanical mixing of liposome and hydrogel using a spatula^77^.

### *In vitro* drug release from LDH incorporated into TCH

*In vitro* drug release study was performed using cellophane dialysis membrane (cut-off 12–14 kDa). The membrane was hydrated with PBS as the receptor medium (pH 5.5) for 12 h. Next, 0.5 g of the liposomal hydrogel containing 0.5 mg DH was suspended into 12 mL of PBS, transferred into dialysis bag as the donor medium, and stirred (37 °C, 120 RPM). Two milliliter aliquots were then taken at the fixed time intervals (0, 60, 120, 180, 240, 300, and 360 min) and were instantly replaced with an equal volume of the fresh buffer. All samples were analyzed for DH content by spectrophotometry at 230 nm. The experiment was carried out in triplicate^78^.

### Stability study

According to the obtained results, TCH provided the most promising results among different hydrogels in terms of mucoadhesion properties and histopathological effects; therefore, from now on, we will consider only TCH in the subsequent experiments.

LDH and LDH incorporated into TCH were stored at 4 °C and 20 °C for 28 days. The mean size of particles in the formulations were examined using SEM at the different time intervals (7, 14, 21, and 28 days) to estimate the effect of different storage temperatures on physical stability of the liposomes^74^.

### Chromatographic system and conditions

The analytical detection of DH was carried out using a Waters Tandem Quadrupole Mass Spectrometer (TQD^TM^), with cooling autosampler and column oven, equipped with electrospray ionization (ESI) interface. The ESI source was set in negative ionization mode as shown in Table 4. An ACQUITY UPLC BEH C18 column (2.1 mm × 50 mm, 1.7 μm) was used for the drug separation using mobile phase of 5 mM ammonium formate and 1% acetonitrile in water (pH 3) as solvent A and 0.1% formic acid in acetonitrile as solvent B with gradient elution at a flow rate of 0.4 mL/min. The gradient began with 80% eluent A and changed linearly to 20% A within 3.5 min at 40 °C. In addition, to control data acquisition, MassLynx V4.1 software with automated data processing using the QuanLynx software was utilized (Waters Corp., Milford, USA)^79^.

**Table 4:** Setting for mass spectra MS/MS detection of DH.

Source (ESI+) and analyzer	Settings
Capillary voltage (kv)	1
Cone voltage (v)	35
Extractor (v)	1
Radio frequency lens (v)	0.2
Source temperature (C)	150
Desolvation temperature (C)	400
Cone gas flow (L/h)	50
Desolvation gas flow (L/h)	800
Collision energy (eV)	27
Collision gas flow (mL/min)	0.25

### Preparation of DH standards and quality control samples

Stock standard solutions of DH and IS from clopidogrel were synthesized in methanol (0.5 mg/mL) and stored in 4-mL amber glass vials at −20 °C. Also, various working standard solutions of DH (0.01–100 µg/mL) and 8 µg/mL of IS were prepared in methanol and freezed in amber vials at −20 °C. In addition, the solutions of plasma standard calibration were prepared in replicate (n = 6) at the drug concentrations of 1, 5, 10, 20, 50 ng/mL. Next, 40 µL of the DH working solutions and 40 µL of the IS were diluted by 200 µL of blank rabbit plasma. Low, medium and high concentration of quality control (QC) samples at the drug concentrations of 1, 10, 50 ng/mL with 100 ng/mL of IS were prepared as well^79^.

### Pharmacokinetics study

Seven New Zealand white rabbits with the mean weight of 3 ± 0.5 kg obtained from KSU Laboratory Animal Facility were used in this study as two groups (n = 3 and 4). All the methods and protocols of animal studies were in accordance with the relevant institutional guidelines and regulations and were approved and permitted by Animal Experimentation Ethics Committee, King Saud University (Riyadh, Saudi Arabia). The animals were housed and fasted for 12 h before the study and had free access to water during the experiment. A cannula was inserted into marginal ear vein for blood sampling and flushed with heparin-normal saline solution. The first group received LDH incorporated into TCH through nasal route and the second group received oral tablet of commercial DH. LDH incorporated into TCH formulation was administered by nasal dropper having a wide orifice inserted 5 g of liposomal hydrogels containing 5 mg of DH into nostrils of the rabbits while they were in supine position. The rabbit was kept in this position for 1 min after administration. A total dose of 5 mg of DH was given to rabbits and 5 mg oral tablet was dispersed in acacia and administered through oral route by dropper^80^.

One milliliter of blood samples collected from cannulated marginal vein before dose administration was considered as a reference level (time point zero) and at time intervals (1, 2, 4, 6 h) in heparinized vacuum glass tubes after administration. The cannula was flushed with heparin in normal saline solution after taking each sample. The plasma was separated by centrifugation method at 1000 RPM for 10 min and stored at −20 °C until the time of analysis^80^.

To precipitate the proteins of the collected plasma samples, 200 µL of aliquoted samples (or calibration standard or QC sample) and 40 µL of IS working solutions were transferred into 1.5-mL Eppendorf tubes, and the mixtures were vortexed for 10 s. Next, 1 mL of acetonitrile was added, and the mixture was vortexed for 1 min, followed by centrifugation at 20,000 RPM for 15 min at 4 °C. The supernatant was transferred into a 1.5-mL Eppendorf tube and evaporated under a gentle stream of nitrogen. The residue was reconstituted in 100 µL of mobile phase, vortexed for 1 min, centrifuged at 4000 RPM for 4 min, and transferred into a plastic autosampler vial with pre-slit septum (Waters, USA). Two microliters of the resultant were then injected into the UPLC MS/MS system^79^.

To evaluate brain concentration of DH, the rabbits’ brain was removed, weighed and homogenized to liquefy the brain tissues. Next, 0.2 µg of the resultant was subjected to the determination of DH brain concentration using UPLC MS/MS system using the above-mentioned protocol^81,82^.

### Data and statistical analysis

The *in vitro* results were shown as the mean ± standard deviation (SD) of triplicate (n = 3), while the *in vivo* results were expressed as mean ± SD of triplicate (for standard curve) and calculated by linear regression without weighing using formula 5:

5 $$Y=a+bX$$

where Y is the ratio of area under peak (AUP) of the drug to the IS, a is intercept, b is slope, and X is the DH concentration.

The pharmacokinetics parameters were calculated using model-independent methods and the terminal elimination rate constant (λ_n_) was estimated using linear regression analysis of the terminal portion of the log-linear blood concentration–time profile of DH. Also, the terminal elimination half-life (t_1/2_) was calculated from the terminal elimination rate constant using formula 6^79^:

6 $${t}_{1/2}=\frac{0.693}{{\lambda }_{n}}$$

The C_max_ and T_max_ were obtained directly from the individual blood levels. The AUCs (µg mL^−1^ h) were measured by the linear trapezoidal rule and extrapolated to time infinity by the addition of C Last/λ_n_, where C Last was the concentration of the last measured plasma sample. The apparent body clearance (Cl/F) was measured using formula 7^79^:

7 $$\frac{Cl}{F}=\frac{Dose}{AUC}$$

The concentration difference at each day was examined using student t-test, and one-way analysis of variance (ANOVA) was applied to evaluate the reproducibility of the assay using IBM SPSS 20 Statistics. The level of confidence was 95%^79^.

## Supplementary information

Supplementary Information
