# Supplementary material for: Nasal delivery of donepezil HCl-loaded hydrogels for the treatment of Alzheimer’s disease
Source: Sci Rep. 2019 Jul 2;9:9563. doi: 10.1038/s41598-019-46032-y (PMC6606601; doi:10.1038/s41598-019-46032-y)
Supplement: Supplementary file 1 — Supplementary Information [file 41598_2019_46032_MOESM1_ESM.docx]

**Nasal delivery of donepezil HCl-loaded hydrogels for the treatment of Alzheimer’s disease**

Sitah Muflih Al Harthi^1,2^, Seyed Ebrahim Alavi^3^, Mahasen Ali Radwan^4^, Mona Mohamed El Khatib^5,6*^ & Ibrahim Abdullah AlSarra^1*^

^1^ Department of Pharmaceutical Science, College of Pharmacy, King Saud University, Riyadh, Saudi Arabia

^2^ Department of Pharmaceutical Science, College of Pharmacy, Shaqra University, Riyadh, Saudi Arabia

^3^ School of Pharmacy, The University of Queensland, Woolloongabba 4102, Australia

^4^ Department of Pharmaceutics and Pharmaceutical Technology, College of Pharmacy, Egyptian Russian University, Bader City, Egypt

^5^ Department of Pharmaceutics, Faculty of Pharmacy, King Saud University, Riyadh, Saudi Arabia

^6^ Department of Pharmaceutics, Faculty of Pharmacy, Cairo University, Cairo, Egypt

*** Corresponding authors:**

Mona Mohamed El Khatib: [mona.elkhatib@pharma.cu.edu.eg](mailto:mona.elkhatib@pharma.cu.edu.eg)

Ibrahim Abdullah AlSarra: [ialsarra@ksu.edu.sa](mailto:ialsarra@ksu.edu.sa)

**Postal address:** Department of Pharmaceutics, Faculty of Pharmacy, King Saud University, Female Section, El-Malaz, PO Box 2457, Riyadh 11451, Saudi Arabia

**Tel:** 00201011352022

**Table 1:** Effect of radiation dose on the gel fraction of PVP hydrogels.

| Radiation dose (KGy) | PVP 3% | PVP 3%+PEG 1% | PVP 3%+PEG 2% | PVP 3%+PEG 3% | PVP 4% | PVP 4%+PEG 1% | PVP 4%+PEG 2% | PVP 4%+PEG 3% | PVP 6% | PVP 6%+PEG 1% | PVP 6%+PEG 2% | PVP 6%+PEG 3% |
| --- | --- | --- | --- | --- | --- | --- | --- | --- | --- | --- | --- | --- |
| 15 | 80% | 79% | 70% | 66% | 94% | 90% | 73.2% | 60% | 97% | 94% | 91.6% | 90% |
| 20 | 82% | 84% | 72% | 70% | 96.4% | 93% | 76% | 66% | 97.8% | 95.4% | 93% | 92% |
| 25 | 85% | 86% | 76% | 74.8 | 97.2% | 96% | 77.6% | 69% | 98% | 97.8% | 95% | 93% |
| 30 | 86% | 88% | 77.4% | 76% | 97.4% | 96.2% | 78.2% | 69.6% | 99% | 97.9% | 96% | 95.4% |

**Table 2:** Swelling percentage of PVP hydrogels.

| Radiation dose (kGy) | PVP 3% | PVP 3%+PEG 1% | PVP 3%+PEG 2% | PVP 3%+PEG 3% | PVP 4% | PVP 4%+PEG 1% | PVP 4%+PEG 2% | PVP 4%+PEG 3% | PVP 6% | PVP 6%+PEG 1% | PVP 6%+PEG 2% | PVP 6%+PEG 3% |
| --- | --- | --- | --- | --- | --- | --- | --- | --- | --- | --- | --- | --- |
| 15 | 20% | 22% | 23% | 23.8% | 19% | 20% | 21.7% | 24% | 14% | 14% | 14.3% | 16% |
| 20 | 18% | 21.3% | 22.5% | 23% | 18.7% | 20% | 21.5% | 20.6% | 12% | 12% | 12% | 14.6% |
| 25 | 16% | 19.5% | 20% | 22.6% | 17.8% | 18% | 19.4% | 19.8% | 10.7% | 10% | 12% | 14% |
| 30 | 16% | 19% | 20% | 21% | 18% | 15% | 19% | 19.4% | 10.4% | 10% | 12% | 14% |

**Table 3:** Flow properties of the various hydrogels.

| Gel formula | Flow behavior | |
| --- | --- | --- |
| PVP 3% | Pseudoplastic | Thixotrophy |
| PVP 3% + PEG 1% | Pseudoplastic | Thixotrophy |
| PVP 3% + PEG 2% | Pseudoplastic | Thixotrophy |
| PVP 3% + PEG 3% | Pseudoplastic | Thixotrophy |
| PVP 4% | Pseudoplastic | Thixotrophy |
| PVP 4% + PEG 1% | Pseudoplastic | Thixotrophy |
| PVP 4% + PEG 2% | Pseudoplastic | Thixotrophy |
| PVP 4% + PEG 3% | Pseudoplastic | Thixotrophy |
| PVP 6% | Over measurement of instrument | |
| PVP 6% + PEG 1% | Over measurement of instrument | |
| PVP 6% + PEG 2% | Dilatants | Rheopexy |
| PVP 6% + PEG 3% | Pseudoplastic | Thixotrophy |
| Chitosan | Pseudoplastic | Thixotrophy |
| Thiolated chitosan | Pseudoplastic | Thixotrophy |

**Table 4:** The effect of contact time and type of the PVP hydrogels on the detachment force (N).

| time (sec) | PVP 3% | PVP 3%+PEG 1% | PVP 3%+PEG 2% | PVP 3%+PEG 3% | PVP 4% | PVP 4%+PEG 1% | PVP 4%+PEG 2% | PVP 4%+PEG 3% | PVP 6% | PVP 6%+PEG 1% | PVP 6%+PEG 2% | PVP 6%+PEG 3% |
| --- | --- | --- | --- | --- | --- | --- | --- | --- | --- | --- | --- | --- |
| 30 | 8 | 9 | 9.8 | 6 | 6 | 7.2 | 8.4 | 6 | 5 | 5 | 5.6 | 6 |
| 120 | 8.5 | 9.6 | 10 | 8.6 | 7.7 | 7.5 | 10.1 | 6.4 | 5.4 | 5.8 | 6 | 9.4 |
| 300 | 9.7 | 9.9 | 10 | 9.5 | 8 | 8.4 | 11 | 7.9 | 7 | 6.8 | 6.3 | 9.8 |
| 600 | 10 | 10 | 11.7 | 9.7 | 8.6 | 8.7 | 11.9 | 9 | 7.8 | 7.7 | 6.8 | 10.4 |

**Table 5:** Effect of contact time on the detachment force (N) of chitosan and thiolated chitosan hydrogels.

| Time (sec) | Chitosan (N) | Thiolated chitosan (N) |
| --- | --- | --- |
| 30 | 5 | 8.3 |
| 120 | 6.5 | 10.7 |
| 300 | 8 | 16.1 |
| 600 | 10 | 20.6 |
